# Surgical repair for giant ascending aortic aneurysm to superior vena cava fistula with positive syphilitic test

**DOI:** 10.1007/s11748-013-0317-2

**Published:** 2013-09-03

**Authors:** Yuji Sekine, Shin Yamamoto, Takuya Fujikawa, Susumu Oshima, Makoto Ono, Shiro Sasaguri

**Affiliations:** Department of Aortic Surgery, Kawasaki Saiwai Hospital, 31-27 Omiyacho, Saiwai ward, Kawasaki, 212-0014 Japan

**Keywords:** Syphilitic aneurysm, Giant aneurysm, Aorta to superior vena cava fistula, Superior vena cava syndrome, Total arch replacement

## Abstract

Syphilitic aortitis is usually associated with thoracic aortic saccular aneurysm, aortic regurgitation and coronary ostial stenosis. However, syphilitic aneurysms have rarely been reported today. Here, we report a patient with ascending aortic aneurysm with aorta-superior vena cava (SVC) fistula with positive syphilitic test. A 52-year-old man was admitted to our institution with a giant ascending aortic aneurysm complicated with SVC syndrome. Computed tomography revealed a giant ascending aneurysm 79 mm in diameter. The result of serodiagnostic tests for syphilis had not been judged yet preoperatively. Total arch replacement concomitant with elephant trunk was performed. Intraoperatively, we detected the ascending aorta to SVC fistula. Postoperatively, we suspected the syphilitic aneurysm strongly, because preoperative serodiagnostic test was concluded to be positive. However, histological examination did not show typical syphilitic features. The patient remains asymptomatic 1 year later. Although extremely rarely today, syphilitic aneurysm should be still considered in the differential diagnosis of ascending aortic aneurysm.

## Introduction

Since the cases of tertiary syphilis can be seen very rarely in Western society today, it is very difficult for us to diagnose as a syphilitic aneurysm preoperatively when we encounter the thoracic aortic aneurysm whose etiology is unknown. Major cardiovascular manifestations of tertiary syphilis are asymptomatic aortitis, aortic regurgitation, coronary ostial stenosis and aortic aneurysm. Here, we report the patient who had suffered from giant ascending aortic aneurysm with positive syphilitic test, moderate aortic regurgitation and fistula between aorta and superior vena cava (SVC).

### Case

**Fig. 1:**
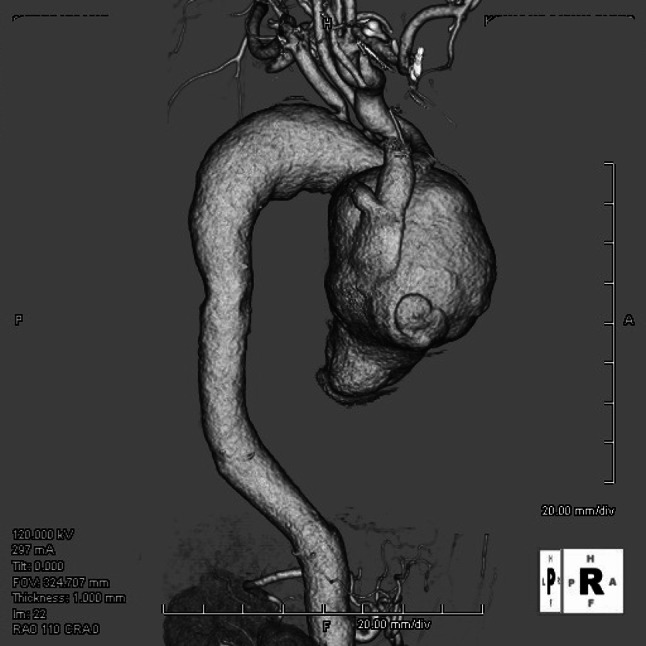
Preoperative computed tomography showing giant ascending aortic aneurysm with the dimension of 79 mm in size and severely compressed SVC

**Fig. 2:**
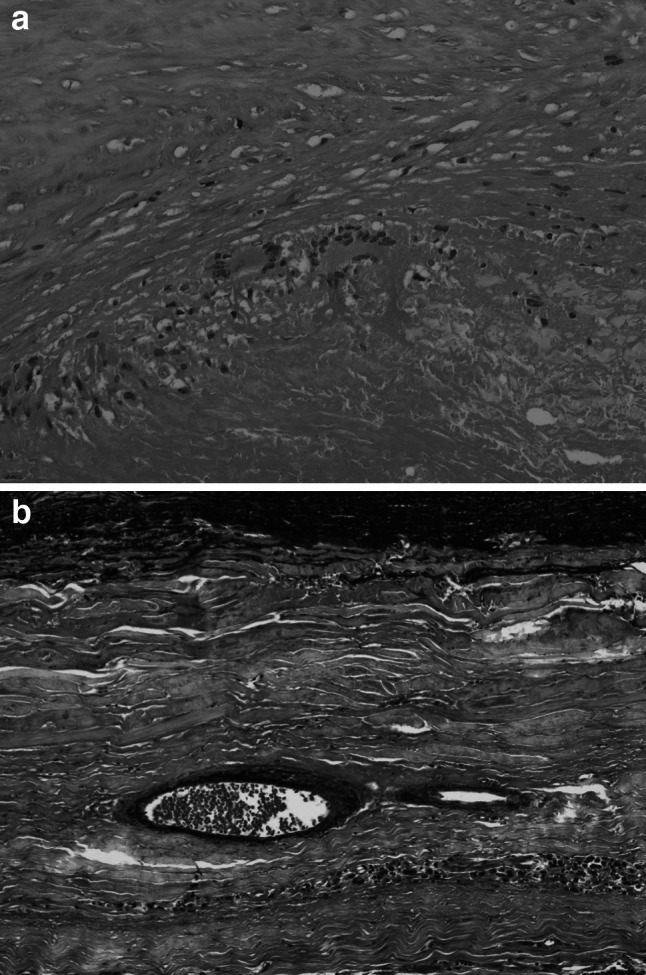
Histological examination. **a** Low-power magnification of tunica media showing medial necrosis with destruction of elastic fibers (H&E staining, ×20). **b** Low-power magnification of adventitia showing no endarteritis obliterans within the adventitia (Elastica–Masson staining, ×10)

A 52-year-old man had suffered from severe edema of his face and upper extremity. He was diagnosed with SVC syndrome and referred to our institution for the treatment of SVC syndrome. Computed tomography scan revealed giant ascending aortic aneurysm with the dimension of 79 mm in size and SVC was compressed severely by ascending aorta (Fig. 1). Echocardiography showed moderate aortic regurgitation and no dilatation of left ventricle. Coronary angiography showed normal coronary arteries including normal coronary ostia. Although we had checked serodiagnostic tests for syphilis, *Treponema pallidum* hemagglutination reaction (TPHA) and fluorescence test assay absorption (FTA-ABS), the results of their examination were not revealed preoperatively. We performed surgical repair of the aortic aneurysm via median sternotomy in a usual manner. However, we detected moderate serous pericardial effusion and severe adhesion around ascending aorta. We could not dissect between ascending aorta and SVC because of severe adhesion. Cardiopulmonary bypass (CPB) was established between bilateral vena cava drainage and right femoral artery perfusion. The patient was cooled to the core temperature of 25 °C, the aneurysm was opened under deep hypothermic circulatory arrest and selective cerebral perfusion was established. Cardiac standstill was established by retrograde and selective cardioplegic infusion. The marked adhesion was detected around distal aortic arch suggesting the inflammatory process around the aorta. The distal anastomosis was performed at 6 cm distal site from left subclavian artery. Since the anastomotic site was slightly dilated, elephant trunk technique was performed simultaneously. After the distal anastomosis under open distal technique, three neck vessels were reconstructed individually. When we started systemic rewarming, we detected the bleeding from the hole of the opened aortic wall. The hole was slit shape and 8 × 1 mm in size. We diagnosed the fistula between ascending aorta and SVC, and closed the fistula with 4-0 polypropylene in a running fashion. The weaning from CPB was uneventful. Postoperatively, we suspected syphilitic aneurysm because we could find that the preoperative serodiagnostic tests were all positive. Although postoperative histological examination of the aneurysmal wall did not show typical syphilitic features, obliterative endarteritis and fibrosis at vasa vasorum, the severely changed tunica media could be detected (Fig. 2a, b). Therefore, we diagnosed syphilitic aneurysm clinically. Penicillin G was administered intravenously for 4 weeks. The patient was discharged from our hospital on postoperative day 45. The patient remains asymptomatic 1 year later.

## Discussion

Syphilis is an infectious disease caused by the spirochete *T. pallidum*. Syphilitic aortitis manifests clinically as aortic regurgitation, coronary ostial stenosis and aortic aneurysm more than 10–20 years after the primary infection [1, 2]. The spirochetal invasion of the aortic adventitia causes an obliterative endarteritis of the vasa vasorum. Blood supply to the aortic wall is impaired, which results in weakening of the tunica media and formation of the aneurysm [3]. In our case, we could not detect typical obliterative endarteritis, but severely changed tunica media could be detected. Atherosclerotic change was only mild. Among syphilitic thoracic aortic aneurysms, saccular aneurysm is more frequent than fusiform. The most frequently involved segment is the ascending aorta, next is the aortic arch and last is the descending aorta. Syphilitic abdominal aortic aneurysm is quite rare [1].

The prognosis for patients with syphilitic aneurysms is extremely poor with the 2-year mortality rate of unrepaired syphilitic aneurysm of 80 %. The majority of death due to aortic aneurysms are caused by rupture [4, 5].

Since cardiovascular syphilitic infection has disappeared in developed countries since 1990, we rarely encounter tertiary syphilis today [6]. But we should consider syphilitic aneurysm in the differential diagnosis of thoracic aneurysms especially when the patient does not have manifestations of connective tissue diseases and has no risk factors for atherosclerosis [7]. We can use serologic tests for screening and diagnosis effectively [6].

Giant syphilitic aortic aneurysm may compress around structures including the trachea, esophagus, pulmonary artery and SVC as in our patient. Although the reports of the arterial fistula to pulmonary artery and the SVC syndrome can be found, our case of the arterial fistula to SVC is first reported case [8–10]. We misdiagnosed as the atherosclerotic aneurysm complicated with simple SVC syndrome when we checked preoperative-enhanced computed tomography.

The most recommended antibiotics is Benzylpenicillin potassium (PCG) of 1.2–2.4 million units daily for 3 weeks [11]. We can use the rapid plasma regain (RPR) as monitoring marker. Although the patient remains asymptomatic 1 and half years later, we should follow the patient carefully for a long time.

## Conclusion

Although syphilitic aortitis has become extremely rare today, syphilis should still be considered as a differential diagnosis of the etiology of aortic aneurysm, especially when the patient does not have manifestations of connective tissue diseases and any risk factors for atherosclerosis.

## References

[1] Jackman JD, Radolf JD (1989). Cardiovascular syphilis. Am J Med.

[2] Roberts WC, Ko JM, Vowels TJ (2009). Natural history of syphilitic aortitis. Am J Cardiol.

[3] Taufiek KR, Chir B, Robert PG (2011). Giant syphilitic aortic aneurysm. N Engl J Med.

[4] Lam AK, Chan AC (1992). Aortic aneurysm at autopsy: a five year survey in Hong Kong. Am J Cardiovasc Pathol.

[5] Torsten B, Battellini R, Kotowicz V, Falk V, Gummert JF, Mohr FW (2004). Ruptured giant syphilitic aneurysm of the descending aorta in an octogenarian. J Card Surg.

[6] Tomey MI, Murthy VL, Beckman JA (2011). Giant syphilitic aortic aneurysm: a case report and review of the literature. Vasc Med.

[7] Umesue M, Durairaj M, Matalanis G, Parsons S (2006). Surgical repair of a syphilitic aneurysm of the distal arch and descending aorta. Asian Cardiovasc Thorac Ann.

[8] Canniere D, Simonart T, Jansens J, Parent D (1999). 21st-century imaging for a 19th-century disease. Circulation.

[9] Phillips PL, Amberson JB, Libby DM (1981). Syphilitic aortic aneurysm presenting with the superior vena cava syndrome. Am J Med.

[10] Pessotto R, Santini F, Bertolini P, Faggian G, Chiominto B, Mazzucco A (1995). Surgical treatment of an aortopulmonary artery fistula complicating a syphilitic aortic aneurysm. Cardiovasc Surg.

[11] Kobayashi T, Yagi T, Murakami M, Jinbo M, Saito S, Takahashi T (2011). Staged surgical repair for extensive cardiovascular damage by syphilis. Ann Thorac Surg.

